# Antenatal corticosteroid administration-to-birth interval and neonatal outcomes in very preterm infants: A secondary analysis based on a prospective cohort study

**DOI:** 10.1371/journal.pone.0281509

**Published:** 2023-02-10

**Authors:** Duan Wang, Li Ming, Yong Zhu

**Affiliations:** 1 Department of Rehabilitation, Children’s Hospital of Chongqing Medical University, Chongqing, China; 2 Department of Pediatrics, Xinqiao Hospital, Army Medical University, Chongqing, China; 3 Department of Pediatric Center, University-Town Hospital of Chongqing Medical University, Chongqing, China; Kasr Alainy Medical School, Cairo University, EGYPT

## Abstract

**Introduction:**

Despite the prevalent use of antenatal corticosteroids (ACS) to prevent preterm infants’ adverse neonatal complications, there is currently no consensus on administration-to-birth intervals of ACS. International guidelines broadly agree that the administration of antenatal corticosteroids should be within 7 days prior to preterm birth. However, there is little evidence to support narrower optimal ACS administration-to-birth interval time. This study was undertaken to investigate the association between the administration-to-birth interval of ACS which is bounded by 48 hours and neonatal outcomes in very preterm infants.

**Materials and methods:**

This is a single-center prospective observational study. Data were collected prospectively from eligible infants from January 2008 to April 2014 at the Santa Clara Valley Medical Center, neonatal outcomes were compared between two groups based on the interval of antenatal corticosteroid administration-to-birth: the interval of <48h, and the interval of >48h. It was noted that the entire study was completed by Dongli Song et al., and uploaded the data to the DATADRYAD website. The author only used this data for secondary analysis.

**Results:**

After adjusting potential confounders (gestational age, sex, birth weight, duration of cord clamping and delivery mode), the interval of >48h group compared to the interval of <48h group had significant reductions in mortality (OR: 0.17; 95% CI: 0.05–0.59), any retinopathy of prematurity (OR: 0.36; 95% CI: 0.16–0.82), severe retinopathy of prematurity (OR: 0.07; 95% CI: 0.01–0.45), any intubation (OR: 0.39; 95% CI: 0.20–0.75) and higher 1 min Apgar (β: 0.56; 95% CI: 0.10–1.02).

**Conclusion:**

This study shows that in very preterm infants, compared with the interval of ACS<48h, the interval of ACS>48 hours has a significant health promotion effect.

## Introduction

Preterm birth is a worldwide epidemic with a global incidence of 15 million every year [1], while rates of preterm birth in the United States have decreased over the last decade, nearly one out of every ten infants is stillborn preterm [2]. Premature infants are more likely to suffer from respiratory distress syndrome, sepsis, intraventricular hemorrhage (IVH), and necrotizing enterocolitis (NEC) shortly after birth [3, 4], preterm infants also face long-term problems including neurodevelopmental delay, retinopathy of prematurity (ROP), chronic lung disease (CLD) and metabolic dysfunction [5, 6].

Antenatal corticosteroids (ACS) has been widely accepted as the standard for managing pregnancies at risk for preterm delivery, and the impact of ACS on reducing the morbidity and mortality of preterm infants has revolutionized perinatal medicine [7]. In preterm infants, ACS have been shown to significantly reduce the risk of adverse complications, including neonatal death, respiratory distress syndrome, IVH, and sepsis, when corticosteroids are given to pregnant women with a higher likelihood of preterm labor before 34 weeks of gestation [8, 9]. Furthermore, there is no evidence that ACS increases the risk of chorioamnionitis, endometritis, or maternal death [9].

Despite the widespread use of ACS to prevent adverse neonatal complications in preterm infants, and several national and international clinical practice guidelines also make recommendations for initial prenatal corticosteroid administration, there is currently no consensus as to the type of corticosteroid to use, the dose and frequency of use, the timing of use and the route of administration [10–14]. International guidelines broadly agree that the administration of antenatal corticosteroids should be within 7 days prior to preterm birth. However, there have been very few studies investigating the interval time within 7 days between ACS administration to birth, and the results of these studies are inconsistent [15–17].

Based on data that has already been published, we conducted a secondary data analysis for this study [18]. The administration-to-birth interval of ACS was used as an independent variable, and the outcome variables and other covariates are consistent with those in the original study. Aim to investigate the association between the administration-to-birth interval of ACS which is bounded by 48 hours and neonatal outcomes in very preterm infants.

## Materials and methods

### Data source

We obtained data from the ‘DATADRYAD’ database (www.Datadryad.org). This website permitted users to freely download the raw data. According to Dryad’s Terms of Service, we cited the Dryad data package in the present study. (Dryad data package: Dongli Song; Jegatheesan, Priya; DeSandre, Glenn; Govindaswami, Balaji (2016), Data from Duration of cord clamping and neonatal outcomes in very preterm infants, Dryad, Dataset, https://doi.org/10.5061/dryad.4q3d3). Variables included in the database file were as follows: birth weight, gestational age (GA), duration of cord clamping(duration of time for the neonate at birth before clamping the umbilical cord), multiple pregnancies, the administration-to-birth interval of ACS (time interval from first ACS dose to delivery, ACS in this study refers to complete and incomplete course of medication, because of the high collinearity between number of doses and the interval of ACS, information of one or more doses was not included, as in previous similar studies), delivery mode (cesarean or vaginal delivery), Apgars at 1 and 5 minutes, delivery room (DR) intubation and positive pressure ventilation, DR chest compression and medications, admission temperature (temperature on NICU admission in Celsius), hypothermia (<36 degree Celsius on admission), hematocrit in the first 2 hours of life, hematocrit between 12–36 hours of life, surfactant administration, pneumothorax, intubation <24hr (intubation within 24 hours of life), any intubation (defined as at least once during NICU stay), any red blood cell transfusion (any red blood cell transfusion during the NICU stay), peak bilirubin (mg/dl), death (death before discharge home), any IVH (any grade of intraventricular hemorrhage, grade 1–4), severe IVH (grade 3 or 4, defined as IVH with ventricular dilatation or parenchymal hemorrhage), late onset sepsis (LOS, the age of onset and timing of the sepsis episode >72 h of life), any ROP (any stage of retinopathy of prematurity, stage 1–5), severe ROP (defined as ≥stage 3 or with Plus disease or requiring surgery or anti-vascular endothelial growth factor treatment for ROP.), NEC, CLD, survival without major morbidity (defined as survival without severe IVH, NEC, CLD, Late Onset Sepsis, or severe ROP).

### Study population

Dongli Song, et al. [18] completed the entire study. We outline the research steps below to provide a clearer understanding of the entire study process. This observational study was conducted from January 2008 to April 2014 at the Santa Clara Valley Medical Center (SCVMC), a safety-net teaching hospital with a high-risk obstetric service and a regional level III Neonatal Intensive Care Unit (NICU). Data were collected prospectively from January 2008. The study included 353 very preterm infants born at < 32 weeks gestation. Exclusion standards: obstetric causes (placental or cord causes, i.e. placental abruption, placenta previa, cord evulsion, and true knot) and fetal or neonatal causes (i.e. severely compromised infant without spontaneous respiration requiring immediate resuscitation after birth). NICU criteria for DR resuscitation, intubation, surfactant therapy, and transfusion and the target oxygen saturation range for preterm infants did not change during the study period. Demographic characteristics, delivery room /NICU practices, neonatal mortality, and morbidities were collected. This original study was approved by the SCVMC institutional review board as a quality improvement project and informed consent was waived.

Our study divided participants into two groups based on the interval of ACS admission: the interval of ACS>48h and the interval of ACS<48h. A total of 10 participants did not use antenatal steroids, and 343 very preterm infants born at < 32 weeks gestation age were included in this study, subgroup study was conducted on extremely preterm infants with gestation age < 28 weeks.

### Statistical analysis

Continuous variables were expressed as mean ± standard deviation, and categorical variables were expressed in frequency or as a percentage. The One-Way ANOVA (normal distribution), Kruskal Wallis H (skewed distribution) test and chi-square tests (categorical variables) were used to determine any statistical differences between the means and proportions of the groups. Fisher Exact for categorical variables with Expects<10. Multivariate logistic regression analysis was used to analyze the significant results between groups, both non-adjusted and multivariate-adjusted models were listed in the paper. According to the recommendation of the STROBE statement, we simultaneously showed the results of unadjusted, minimally adjusted analyses and those fully adjusted analyses. Adjusted covariates include gestational age, sex, birth weight, Duration of Cord Clamping (DCC) group (the 30-45s DCC group and the 60-75s DCC group), and delivery mode. Due to the small proportion of missing values, cases with missing data were deleted during the analysis. All of the analyses were performed with the statistical software packages R (http://www.R-project.org, The R Foundation) and EmpowerStats (http://www.empowerstats.com, X&Y Solutions, Inc., Boston, MA). P values less than 0.05 (two-sided) were considered statistically significant.

## Results

### Demographic characteristics of participants

343 very preterm infants born at < 32 weeks gestation age were included in this study. The average gestational age of the participants was 29.7±2.6 weeks, and approximately 38.8% of them were female. Multiple pregnancies accounted for 19.8% of the study participants and cesarean section accounted for 61.2%. Demographics of the study patients are shown in Table 1. There were no significant differences between the two intervals of ACS groups of preterm infants. Of all participants, 2 of them had a lack of peak bilirubin, 16 had a lack of any ROP and severe ROP, and 17 had a lack of CLD outcome.

**Table 1:** Baseline characteristics of participants (N = 343)^a^.

	ACS		
Characteristic	<48hours prior to delivery	>48hours prior to delivery	P-value
**No of participants**	105	238	
**Birth weight (g)**	1390.4 ± 461.4	1380.0 ± 460.8	0.847
**Gestational age (week)**	29.5 ± 2.6	29.8 ± 2.5	0.408
**Duration of cord clamping(%)**			0.890
30-45s	56 (53.3%)	125 (52.5%)	
60-75s	49 (46.7%)	113 (47.5%)	
**Sex (%)**			0.635
Female	43 (41.0%)	91 (38.2%)	
Male	62 (59.0%)	147 (61.8%)	
**Multiple pregnancy(%)**			0.157
No	89 (84.8%)	186 (78.2%)	
Yes	16 (15.2%)	52 (21.8%)	
**Vaginal delivery(%)**			0.080
No	57 (54.3%)	153 (64.3%)	
Yes	48 (45.7%)	85 (35.7%)	

^a^Values are means ± SD or n(%) SD = standard deviation.

### Univariate analysis

We compared the differences in the interval of ACS and neonatal outcomes between preterm infants at <32 weeks and <28 weeks of gestational age. The results of the univariate analysis are shown in Table 2. About the delivery Room Measures, in the GA <32 weeks group, the 1 min Apgar was higher in the interval of the ACS>48h group than in the interval of the ACS<48h group (6.2±2.1 vs. 5.7±2.4). Likewise, in the GA <28 weeks (extremely preterm birth) subgroup, the 5 min Apgar score was significantly higher in the interval of ACS>48h group (6.9±1.7 vs. 6.1±2.0). And any red blood cell infusion during hospitalization at the NICU was significantly lower (92.3% vs. 70.8%). Other DR and NICU measurements did not differ between the two ACS groups.

**Table 2:** Neonatal outcomes in very preterm infants^a^.

	<32weeks at GA			<28weeks at GA		
Characteristic	<48hours prior to delivery(n = 105)	>48hours prior to delivery(n = 238)	P-value	<48hours prior to delivery(n = 26)	>48hours prior to delivery(n = 65)	P-value
**Delivery Room Measures**						
1 min Apgar	5.7 ± 2.4	6.2 ± 2.1	**0.034**	4.3 ± 2.5	5.2 ± 2.3	0.077
5 min Apgar	7.4 ± 1.7	7.7 ± 1.6	0.110	6.1 ± 2.0	6.9 ± 1.7	**0.046**
DR Intubation	21 (20.0%)	34 (14.3%)	0.184	15 (57.7%)	24 (36.9%)	0.071
DR Chest Compression and Medications	4 (3.8%)	5 (2.1%)	0.362	1 (3.8%)	3 (4.6%)	1.000
Admission Temperature <36°C	3 (2.9%)	7 (2.9%)	0.966	0 (0.0%)	1 (1.5%)	1.000
**Respiratory Measures**						
Surfactant, %	25 (23.8%)	45 (18.9%)	0.299	16 (61.5%)	33 (50.8%)	0.352
Intubation <24hr	33 (31.4%)	57 (23.9%)	0.147	20 (76.9%)	40 (61.5%)	0.162
Any Intubation	42 (40.0%)	71 (29.8%)	0.065	21 (80.8%)	49 (75.4%)	0.582
Pneumothorax	5 (4.8%)	7 (2.9%)	0.398	4 (15.4%)	6 (9.2%)	0.396
**Hematological Measures**						
Peak Bilirubin (mg/dL)	8.3 ± 2.8	8.5 ± 2.5	0.604	5.7 ± 1.8	6.2 ± 1.8	0.207
Any Red Blood Cell Transfusion	32 (30.5%)	60 (25.2%)	0.310	24 (92.3%)	46 (70.8%)	**0.028**
<2h Hct	47.3 ± 6.9	48.6 ± 7.0	0.108	42.0 ± 4.1	43.6 ± 6.5	0.244
12-36h Hct	49.1 ± 8.7	51.1 ± 8.0	0.069	42.8 ± 6.6	45.4 ± 8.6	0.175
**Neonatal Mortality and Morbidities**						
Death	10 (9.5%)	5 (2.1%)	**0.002**	7 (26.9%)	5 (7.7%)	**0.014**
Survival without major morbidity	74 (70.5%)	183 (76.9%)	0.207	6 (23.1%)	22 (33.8%)	0.315
Any IVH	27 (25.7%)	39 (16.4%)	**0.043**	15 (57.7%)	26 (40.0%)	0.125
Severe IVH	10 (9.5%)	11 (4.6%)	0.081	9 (34.6%)	10 (15.4%)	**0.041**
CLD	12 (12.6%)	30 (12.9%)	0.952	9 (47.4%)	26 (43.3%)	0.758
Late Onset Sepsis	5 (4.8%)	16 (6.7%)	0.485	5 (19.2%)	11 (16.9%)	0.794
Any ROP	27 (28.4%)	51 (21.8%)	0.200	15 (78.9%)	35 (57.4%)	0.090
Severe ROP	8 (8.4%)	7 (3.0%)	**0.032**	7 (36.8%)	7 (11.5%)	**0.011**
Any NEC	4 (3.8%)	11 (4.6%)	0.735	2 (7.7%)	9 (13.8%)	0.416

^a^Values are means ± SDs or n(%) SD = standard deviation.

In terms of neonatal mortality, compared with the interval of the ACS<48h group, the interval of the ACS>48h group had significantly lower mortality (9.5% vs. 2.1%). A similar reduction was observed in the <28-week GA subgroup (26.9% vs. 7.7%). Regarding neonatal morbidity, any IVH, severe IVH, and severe ROP were significantly lower in the interval of ACS>48h group. A similar reduction was observed in the < 28-week GA subgroup except for any IVH, in addition, in the < 28-week GA subgroup, the mortality of severe ROP also decreased significantly in the interval of ACS>48h group. There were no differences in survival without major morbidity, CLD, LOS, and NEC between the two ACS groups.

### The relationship between the interval of ACS and neonatal outcomes

As shown in Table 3, after adjusting for GA, sex, birth weight, DCC group, delivery mode, multivariate logistic regression revealed that five candidate factors remained significant: the observed mortality (OR = 0.17; 95%CI = 0.05–0.59), any ROP (OR = 0.36; 95% CI = 0.16–0.82), severe ROP (OR = 0.07; 95% CI = 0.01–0.45), 1 min Apgar (β = 0.56; 95% CI = 0.10–1.02) and any intubation (OR = 0.39; 95% CI = 0.20–0.75). In the <28-week GA subgroup, significant reductions in mortality (OR = 0.20; 95% CI = 0.04–0.95) and severe ROP (OR = 0.06; 95% CI = 0.01–0.58) were also observed after risk adjustment. There were no statistically significant differences in any ROP, 1min Apgar, and intubation in the GA<28 weeks subgroup after final adjustments.

**Table 3:** Multivariate logistic regression analysis of neonatal outcomes.

	<32weeks at GA			<28weeks at GA		
Outcome	Model I	Model II	Model III	Model I^a^	Model II^a^	Model III^a^
Death	**0.20 (0.07, 0.61) 0.005**	**0.19 (0.06, 0.63)0.007**	**0.17 (0.05, 0.59)0.005**	**0.23 (0.06, 0.80) 0.021**	**0.23 (0.06, 0.84) 0.026**	**0.20 (0.04, 0.95) 0.042**
Any IVH	**0.57 (0.32, 0.99) 0.045**	0.60 (0.31, 1.16) 0.127	0.59 (0.30, 1.15) 0.121	0.49 (0.19, 1.23) 0.129	0.55 (0.20, 1.47) 0.233	0.76 (0.27, 2.13) 0.597
Severe IVH	0.46 (0.19, 1.12) 0.087	0.47 (0.16, 1.36)0.164	0.45 (0.15, 1.29) 0.137	**0.34 (0.12, 0.98) 0.047**	0.35 (0.12, 1.03) 0.056	0.39 (0.12, 1.27) 0.120
Any ROP	0.70 (0.41, 1.21) 0.202	**0.45 (0.21, 0.97) 0.041**	**0.36 (0.16, 0.82) 0.014**	0.36 (0.11, 1.21) 0.098	0.28 (0.08, 1.02) 0.053	0.29 (0.07, 1.27) 0.100
Severe ROP	**0.34 (0.12, 0.95) 0.040**	**0.12 (0.02, 0.57) 0.008**	**0.07 (0.01, 0.45) 0.006**	**0.22 (0.07, 0.75) 0.016**	**0.10 (0.02, 0.46) 0.003**	**0.06 (0.01, 0.58) 0.015**
1 min Apgar^b^	**0.54 (0.04, 1.05) 0.034**	**0.54 (0.08, 1.00) 0.022**	**0.56 (0.10, 1.02) 0.018**	0.98 (-0.09, 2.05) 0.077	0.97 (-0.13, 2.07) 0.086	0.54 (-0.50, 1.58) 0.308
5 min Apgar^b^	0.30 (-0.07, 0.67) 0.110	0.27 (-0.06, 0.61) 0.114	0.28 (-0.06, 0.62) 0.108	0.83 (0.03, 1.64) 0.046	0.75 (-0.07, 1.58) 0.076	0.53 (-0.28, 1.33) 0.203
Any Intubation	0.64 (0.39, 1.03) 0.066	**0.41 (0.22, 0.78) 0.007**	**0.39 (0.20, 0.75) 0.005**	0.73 (0.24, 2.25) 0.583	0.62 (0.19, 2.01) 0.424	1.34 (0.31, 5.86) 0.695
Any Red Blood Cell Transfusion	0.77 (0.46, 1.28) 0.311	0.53 (0.24, 1.13) 0.101	0.50 (0.23, 1.12) 0.093	0.20 (0.04, 0.94) 0.041	0.15 (0.03, 0.75) 0.021	0.18 (0.03, 1.11) 0.064

Values are OR/β(95%CI) CI = confidence interval. OR = odds ratio.

Model I adjust for: None.

Model II adjust for: GA; sex; delivery mode.

Model III adjust for:GA; sex; birth weight; DCC group; delivery mode.

^a^ Model not adjusted for GA.

^b^ Analysis as a continuous variable.

## Discussion

The administration of ACS to reduce morbidity and mortality in newborns at risk of preterm birth before 34–35 weeks of gestational age has been widely accepted and it is now firmly established by guidelines across the world as an evidence-based suggestion [9, 13, 19]. Although fetal lung maturation is generally seen as the primary benefit of ACS treatment, it has also been linked to additional advantages in preterm infants.

In the present study, we found a clear association between the interval of ACS and survival in very preterm infants, the mortality of very preterm infants with an interval of ACS>48h was significantly decreased compared with those with the interval of ACS<48h, as well as in extremely preterm birth. We also found that infants receiving the interval of ACS>48h required less respiratory intervention, had higher 1min Apgar and had a significantly lower risk of ROP.

According to most international guideline recommendations, prenatal corticosteroids should be administered within seven days before a premature birth [13, 19, 20]. However, there is little evidence to support narrower optimal ACS administration-to-birth interval time. Some animal experiments have shown that ACS administration > 48 hours before delivery shows greater benefits in surfactant production than 24 hours before delivery [21, 22], and some vitro studies suggested that the effects of dexamethasone on surfactant protein B gene transcription were maximal at 48 hours of the initial exposure [23, 24]. In a twin pregnancy study, It was only when the ACS delivery interval was between 2 and 7 days that the ACS was associated with a lower risk of RDS [25]. Likewise, Gulersen M found that the adverse outcomes were more common in those born with the ACS interval less than 2 days than in those born with the ACS interval 2–7 days [26]. Antenatal corticosteroids may have positive benefits as early as 3 hours after administration, with their greatest mortality-reducing effects occurring 18 to 36 hours later, according to recent data from the Effective Perinatal Care in Europe cohort research, in addition, 48 hours to 1-week interval of ACS was associated with a significantly lower incidence of serious neonatal brain injury (defined as an intraventricular hemorrhage grade of 3 or 4) [27].

In terms of neonatal mortality, our results are consistent with these findings, in addition, due to the beneficial effects of ACS on the respiratory and cardiovascular systems [10, 28], higher 1 min Apgar scores and lower any intubation rates were found in the potentially better ACS interval group, but for chronic lung disease, there was no significant difference between the two groups.

An important finding in our study was that the interval of ACS>48 hours was associated with a significant reduction in ROP, at present, there is a lack of studies focusing on the interval of ACS and ROP in preterm infants. The retina of a full-term infant is nearly complete at delivery, but the retina of a premature infant is immature at birth [29], the incidence of ROP has strongly connected to gestational age and birth weight, with preterm infants born with low gestational age or birth weight having a higher incidence of ROP [30], our study showed that the association remained significant after adjusting for these factors. Some studies have shown that infants born extremely preterm and with very low birth weight have a decreased risk of severe ROP after ACS [31, 32]. However, a recent meta-analysis suggests that ACS treatment may decrease, but not prevent, the severity of ROP [33]. At present, the evidence of the effect of ACS on ROP is based on some retrospective studies, and more rigorously designed RCT experiments are needed to improve the level of evidence. Meanwhile, our study is a single-center observational study with a relatively limited sample size and the results need to be interpreted with caution. The RCT studies with a larger sample are required to identify the GA-dependent optimal interval of ACS.

Our study is a secondary study of the effect of ACS interval on neonatal outcomes in preterm infants, the interval of ACS was prospectively collected, and the sample size was adequate for analyses that adjusted for mortality and neonatal outcomes according to the interval of ACS and a number of potential confounding variables, and the results were stable and relevant to the management of pregnancies at risk for preterm delivery. However, it has limitations as a single center observational study. First, the original study did not include severely compromised infants who did not have spontaneous respiration and needed resuscitation immediately, some of these preterm infants were already treated for ACS, so the exclusion may have introduced some bias. Second, this observational study itself includes unavoidable possible confounding elements, so in order to reduce these biases, we strictly apply statistical correction to reduce residual confounding. Third, due to raw data limitations, we can not obtain the specific time interval of prenatal exposure to ACS, so the group with ACS > 48 hours was mixed with people with ACS>1 week, some cohort studies have found that increased mortality and adverse outcomes with an ACS administration-to-birth interval exceeding 7 days [17, 27, 34–36], therefore, our conclusions do not apply to preterm infants with ACS > 1 week, after removing these confounders, a stronger association between the interval of ACS>48h and beneficial neonatal outcomes may be found. In view of the fact that this study is a single-center non-RCT study with relatively unitary demographic characteristics, the study has some limitations, moreover, other and unknown differences in case-mix between the different ACS intervals may have occurred, it may be not generalizable to other biographic ethnic groups.

In conclusion, this study shows that in very preterm infants, compared with the interval of ACS<48h, the interval of ACS>48 hours has a significant health promotion effect, this finding is important for clinicians when managing very preterm infants, which may help reduce infant mortality and retinopathy of prematurity.

## Supporting information

S1 Data (XLSX)
